# Four-Vessel Umbilical Cord: Supernumerary Right Umbilical Vein With No Associated Congenital Anomalies

**DOI:** 10.7759/cureus.67283

**Published:** 2024-08-20

**Authors:** McKenzie K Allen, Oletha Minto

**Affiliations:** 1 Gastroenterology, Edward Via College of Osteopathic Medicine, Aiken, USA; 2 Obstetrics and Gynecology, Aiken Obstetrics and Gynecology Associates, Aiken, USA

**Keywords:** four-vessel cord with no associated findings, ultrasound perinatal, antenatal care, congenital anomalies associated with abnormal vessels in umbilical cord, antenatal management, 4-vessel umbilical cord, supernumerary umbilical vein

## Abstract

A four-vessel umbilical cord is a rare anomaly that can occur with abnormal persistence of the caudal portion of the vessel. Although supernumerary vessels can present as an isolated finding, they are known to be associated with multiple significant congenital anomalies. Ectopia cordis, pulmonary stenosis, cleft lip, cleft palate, situs inversus, tetralogy of Fallot, and gastroschisis are some anomalies associated with four-vessel cords. This is a case of a 22-year-old multigravida with a four-vessel umbilical cord initially found on sonography. The patient was sent to Maternal Fetal Medicine for evaluation. It was determined that the patient had a right supernumerary umbilical vein that did not require further workup. The patient presented to labor and delivery at 36 weeks and five days with regular contractions. After normal vaginal delivery without complications, the four-vessel-umbilical cord was visualized and confirmed by pathology. The patient and neonate both did well with no complications.

## Introduction

During gestation, the umbilical cord is a vital lifeline between the placenta and the developing fetus. Assuming normal development, the two umbilical arteries carry deoxygenated fetal blood and carbon dioxide to the placenta. A single umbilical vein transports oxygenated blood and glucose from the placenta to the fetal heart. During week two of development, the extraembryonic mesoderm forms a structure known as the body stalk. Beginning at week three, the vitelline duct forms from the endodermal derivative, the gut tube creating an opening from the midgut to the yolk sac. The hindgut forms the allantois, which grows toward the umbilical cord and functions to drain the bladder. The body stalk, vitelline duct, and allantois are the three major structures that form the umbilical cord. The allantois contains the umbilical arteries and umbilical vein that support fetal oxygenation and circulation throughout pregnancy. There is initially a right and a left umbilical vein. The right umbilical vein typically becomes atretic around the fourth week and is gone by the seventh week [1,2]. The vessels within the umbilical cord facilitate the exchange of nutrients, oxygen, and waste products between the fetus and maternal circulation, ensuring normal growth and development.

A supernumerary umbilical vessel is a rare developmental anomaly that occurs during embryogenesis. There have been reports of umbilical cords with only one vessel and reports of several vessels in addition to the typical two arteries and one vein, all with a wide range of outcomes. For this reason, the clinical significance of supernumerary vessels has not been established [3]. Congenital heart defects are one of the most commonly associated anomalies with the persistence of a supernumerary right umbilical vein [2]. Case reports of four-vessel cords have reported cases of holoprosencephaly, polyhydramnios, omphalocele, and aneuploidies [3]. 

Cases of an isolated extra umbilical vessel with no associated abnormalities have been commonly reported [4,5]. The correlation between four-vessel umbilical cord and congenital abnormalities continues to be a subject of ongoing investigation in the medical community.

The incidence of persistent right umbilical vein is reported to be one in 250 to one in 1,250 [6]. Current research suggests that multiple factors may contribute to abnormalities in the number of vessels within an umbilical cord, such as genetic predispositions, fetal development, and environmental factors that may influence development [4]. No clear causal relationships have been established regarding the occurrence of four-vessel umbilical cords, and further studies are needed to understand this phenomenon fully.

## Case presentation

The patient is a 22-year-old multigravida female, with a past medical history of rheumatoid arthritis, glomerulonephritis, and a short cervix in her previous pregnancy. The patient's history includes one previous vaginal delivery at 39 weeks and five days that was complicated by uterine prolapse. Past abdominal surgical history includes laparoscopic cholecystectomy in 2019. Medications included sertraline 50 mg once daily and a prenatal vitamin. The patient stated that she quit vaping when she found out she was pregnant. A possible supernumerary vessel in the umbilical cord was identified during a routine antenatal ultrasound. The pregnancy had been uncomplicated to this point, and the patient had regular, scheduled prenatal care visits. The patient was sent to Maternal Fetal Medicine. Maternal Fetal Medicine specialists evaluated the patient and reported no additional abnormalities were found; further work-up was not indicated at the time. The patient was monitored with serial ultrasounds throughout the remainder of her pregnancy to evaluate fetal anatomy for any abnormalities. The patient also received weekly biophysical profiles (BPP) and biweekly non-stress tests (NST) beginning at 32 weeks. There were no abnormalities or reasons for concern.

The patient presented to labor and delivery at 36 weeks and five days. At the time, she was 3 cm dilated and having regular contractions occurring every five minutes. The patient reported that she had been leaking clear fluid since the day prior but denied any associated bleeding or pain. The patient reported that she went to another hospital the day prior for contractions and was told she was 2 cm and should go home until contractions became more regular. The patient was admitted for expectant management. Labs at the time were within normal limits. Fetal heart tones were category 1. The nitrazine test was negative for rupture of membranes. At the next check, she was 4 cm, 80% effaced, and at 0 station. The plan was to observe the patient for change. The patient was counseled on the risks, benefits, and options regarding steroid administration and chose to receive Celestone. Ampicillin was ordered because the patient’s Group B Strep testing result was not yet known. The patient was observed for the remainder of the day and overnight and did not progress. She was discharged home and counseled on what circumstances she should come to labor and delivery. 

**Figure 1:**
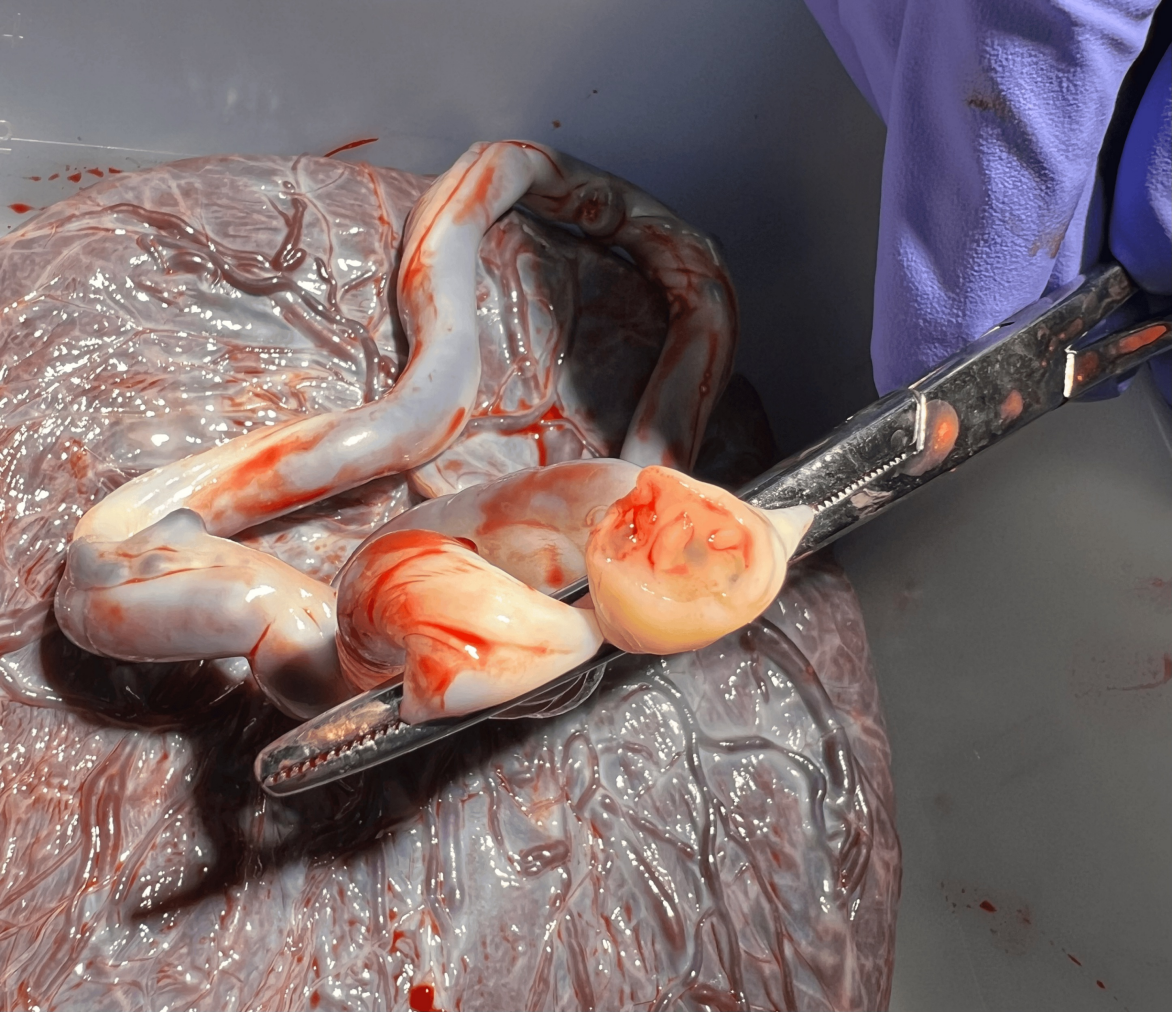
Photograph of a four-vessel umbilical cord, showing two arteries and two veins, immediately after delivery. The umbilical cord is clamped and cut, revealing the distinct vessels.

**Figure 2:**
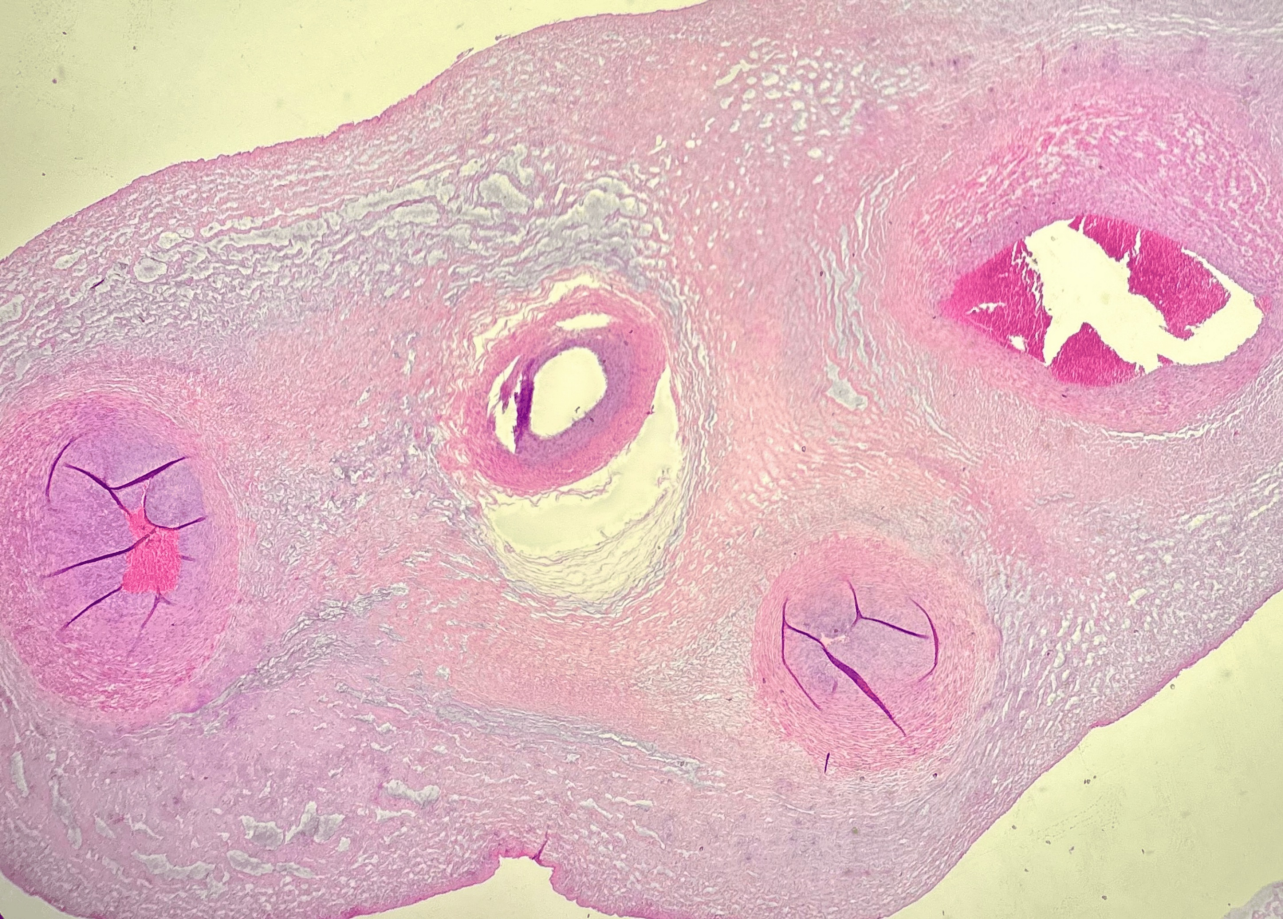
The histological section of the four-vessel umbilical cord is stained with hematoxylin and eosin. It shows two umbilical arteries (the two lower vessels) and two umbilical veins (the two upper vessels). The cross-sectional view highlights the distinct vascular structures within the umbilical cord.

The patient returned two days later with regular, painful contractions, and she was admitted. The patient was 37 weeks at the time. An amniotomy was performed, and the patient received an epidural. The plan was to continue to observe with expectant management until fully dilated. Several hours later, the patient had a normal spontaneous vaginal delivery of a 2,360 g female at 10:45 AM. APGARs were 9 at one minute and 10 at five minutes. The placenta was then delivered and found to be complete and intact. A visible right umbilical vein was present, with four vessels in the umbilical cord (Figure 1). Blood loss was estimated to be 50 mL. The patient and neonate both did well in recovery with no concerns. They were counseled on follow-up and care instructions and were discharged the following day. The placenta was taken to pathology for routine evaluation. Histological staining with hematoxylin and eosin confirmed the presence of a four-vessel umbilical cord with a right supernumerary umbilical vein (Figure 2).

## Discussion

As observed in this case, the presence of a four-vessel umbilical cord underscores the variability and unfamiliarity surrounding these rare anomalies. The umbilical cord most often contains two umbilical arteries and one umbilical vein. The right umbilical vein normally involutes during development. The discovery of an additional vein raises clinical considerations, given the association with a wide array of congenital anomalies [1].

In this case, the supernumerary vessel was identified during routine sonography. Maternal Fetal Medicine specialists evaluated the patient and determined that no additional workup was needed. The patient underwent regular prenatal monitoring, including serial ultrasounds, biophysical profiles, and non-stress tests, all within normal limits, and did not raise any further concerns.

The literature suggests that supernumerary vessels are often linked to severe congenital conditions, such as congenital heart defects, ectopia cordis, and tetralogy of Fallot [2,3]. However, isolated cases without associated abnormalities have been reported, as in this case [4,5]. Individualized assessment of patients with abnormal vessels in their umbilical cord is necessary to identify additional anomalies and allow proactive treatment approaches to increase the likelihood of favorable prognoses for these patients [5].

The clinical management of this patient included shared decision-making with the patient, resulting in the administration of Celestone to support fetal lung maturity during her first hospital admission. Close monitoring during labor and delivery was appropriate and aligned with the standard obstetric practices for cases presenting with potential umbilical cord anomalies [4,5]. The successful full-term delivery with no complications for the mother or neonate demonstrates that careful monitoring and proactive management of such cases can lead to positive outcomes.

## Conclusions

This case report describes a rare occurrence of a four-vessel umbilical cord identified in a 22-year-old multigravida patient. Despite the known associations between anomalous number of umbilical vessels and congenital anomalies, no additional abnormalities were found in this patient or her neonate. This patient received routine antenatal care and had a spontaneous vaginal delivery of a healthy neonate at 37 weeks with no complications.

The findings in this case contribute to the existing knowledge on umbilical cord anomalies and illustrate the need for thorough prenatal evaluation and monitoring in these cases. Further research is needed to understand better the etiology, implications, and optimal management strategies for pregnancies complicated by umbilical cord anomalies. This case exemplifies the potential for favorable maternal and neonatal outcomes with supportive management in patients with abnormal vessels in their umbilical cord.
